# Associations of combined physical activity and dietary quality with all-cause and cardiovascular disease mortality among US adults with chronic kidney disease

**DOI:** 10.1080/0886022X.2024.2437120

**Published:** 2024-12-10

**Authors:** Wenting Zhu, Qiushi Liu, Lihua Zhang, Chenfeng Jiao, Honglang Xie

**Affiliations:** a National Clinical Research Center of Kidney Diseases, Jinling Hospital, Nanjing Medical University, Nanjing, China; b The Department of Urology, The First Affiliated Hospital of Anhui Medical University, Hefei, China; c Institute of Urology & Anhui Province Key Laboratory of Genitourinary Diseases, Anhui Medical University, Hefei, China

**Keywords:** Physical activity, dietary quality, chronic kidney disease, mortality

## Abstract

**Background:**

There is a lack of sufficient information on the impact of physical activity (PA) and dietary quality (DQ) on mortality in patients with chronic kidney disease (CKD), and no study has yet examined the relationship between the combined effects of PA and DQ on the risk of death in patients with CKD in a representative adult population.

**Methods:**

Adult CKD patients (*n* = 6,504) from the National Health and Nutrition Examination Survey (NHANES) 1999–2018 were included in the study. Mortality outcomes were assessed by National Death Index records before 2/25/2019. Four lifestyle categories were established: low-PA individuals with unhealthy diets, low-PA individuals with healthy diets, high-PA individuals with unhealthy diets, and high-PA individuals with healthy diets. Cox proportional risk modeling was used to calculate the hazard ratios (HRs) and 95% confidence intervals (CIs) of various lifestyle categories for all-cause and CVD mortality.

**Results:**

During a median follow-up period of 111 months, 1,971 participants with CKD died from all-cause mortality, and 567 died from CVD among 6,504 respondents. The high-PA CKD population with a healthier diet had a significantly lower risk of all-cause [0.75, 95% CI (0.64–0.87)] and CVD [0.69, 95% CI (0.51–0.93)] mortality than the low-PA, unhealthy diet participants did. The age and race subgroups showed significant interactions, with the older (≥60 years) and non-Hispanic black subgroups experiencing a more favorable risk-lowering effect for all-cause death.

**Conclusion:**

CKD patients with healthy diets and adequate PA had lower risks of CVD and all-cause mortality than did low-PA individuals with unhealthy diets.

## Introduction

Chronic kidney disease (CKD) is a global public health problem, with approximately 13.4% of the world’s population suffering from CKD. By 2036, more than 4 million individuals are expected to have stage 3–5 CKD [1]. Cardiovascular disease (CVD) is a major risk factor for patients with CKD, and this risk is increased two- to fourfold by reduced kidney function and increased urine albumin concentrations [2]. In turn, studies of cohorts with chronic kidney insufficiency have shown that CVD speeds up the rate at which CKD progresses [3]. Therefore, it is crucial to identify modifiable factors to reduce the number of premature deaths related to CKD.

Lifestyle changes (including physical activity, diet quality) play an important role in the management of CKD [4]. During the progression of CKD, there are significant changes in the requirements and utilization of different nutrients. These changes put patients at higher risk for nutritional and metabolic abnormalities that are directly associated with CKD progression and risk of death. However, nutrient-based dietary restrictions are difficult to implement and may result in patients consuming less healthy diets [5]. Overall dietary quality (DQ) is a comprehensive assessment of a person’s entire eating pattern. Healthy eating patterns that emphasize food combinations may be easier for patients to follow and effective in preventing adverse health outcomes. In recent years, a correlation has been proposed between healthy dietary patterns and higher survival rates in CKD patients [6]. A minimum of 150 min of moderate physical activity (PA) per week is recommended for individuals with CKD by the Global Clinical Practice Guidelines for Improving the Evaluation and Management of Lifestyle in CKD for Kidney Disease Outcomes [7]. However, the conclusions are constrained by the consistency of the studies on the benefits of PA on outcomes for those with CKD [8]. Following PA guidelines and reducing sedentary time were shown to reduce all-cause and cause-specific mortality as well as prevent disease progression in various ways in a survey conducted in the US population between 2007–08 and 2017–18[9]. A recent meta-analysis revealed no correlation between exercise and kidney outcomes or all-cause mortality in self-management interventions for CKD [10].

The majority of studies that have been published have focused on the individual effects of DQ or PA and have not combined the two to examine their relationship with both CVD and all-cause mortality in people with CKD. In addition, information related to race may not be available in previous studies, and the impact of racial or ethnic differences on diet is often overlooked. We evaluated the relationships of DQ and exercise with CVD and all-cause mortality in a CKD population *via* continuous NHANES data to close these knowledge gaps.

## Methods

### Study population

A nationally representative study, the National Health and Nutrition Examination Survey (NHANES) was created to evaluate the nutritional status and general health of the noninstitutionalized civilian population in the United States (U.S). The Centers for Disease Control and Prevention’s (CDC) National Center for Health Statistics (NCHS) conducted the NHANES with institutional review board approval. Written informed consent was acquired from each participant.

**Figure 1.**
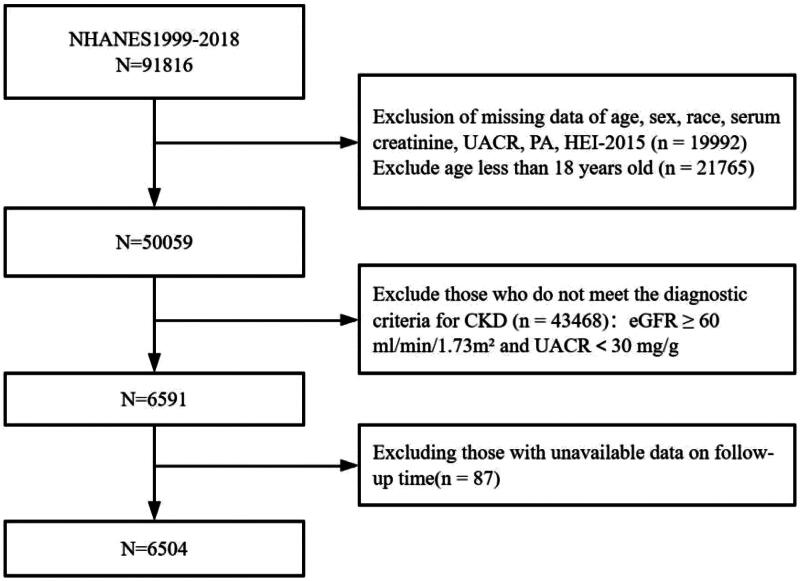
The flowchart of selecting participants. CKD: chronic kidney disease; eGFR: estimated glomerular filtration rate; UACR: urinary albumin to creatinine ratio; PA: physical activity; HEI: Healthy Eating Index.

We used data from 10 NHANES cycles between 1999 and 2018 for the survey. A total of 91,816 participants were extracted. Participants lacking demographic information (age, sex, race/ethnicity), serum creatinine, urinary albumin to creatinine ratio (UACR), PA information, and HEI-2015 information were excluded (*n* = 19,992), and those younger than 18 years of age were excluded (*n* = 21,765). CKD was defined as a UACR of ≥30 mg/g and/or an estimated glomerular filtration rate (eGFR) of <60 mL/min/1.73 m^2^. The eGFR was calculated using the CKD Epidemiology Collaboration’s serum creatinine equation [11]. Based on this criterion, 43,468 individuals without CKD were excluded. In addition, we excluded participants who had no follow-up time (*n* = 87). This study ultimately included 6,504 participants (Figure 1).

### Assessment of the DQ and PA

Two dietary recalls of all food and beverages that were consumed over a 24-h period and separated by three to ten days were used to determine the quality of the diet. An indicator of food quality that is unaffected by quantity, the Healthy Eating Index (HEI)-2015, can be used to evaluate American adherence to the Dietary Guidelines for Americans (DGA) [12]. Thirteen nutrient- and food-based components make up the HEI-2015. There are four ‘moderate’ components (refined grains, sodium, added sugars, and saturated fats) and nine ‘adequate’ food components (total fruits, whole fruits, total vegetables, greens and legumes, whole grains, dairy, total protein foods, seafood and plant proteins, and fatty acids) among the thirteen. The HEI-2015 score ranges from 0 to 100. Higher total scores indicate greater overall DQ [12]. Individuals who achieved a 2-day average score on the HEI that was at least the 60th percentile were deemed to be following recommended dietary guidelines or eating a healthy diet [13].

Data on PA were gathered using the Global PA Questionnaire. Three types of PAs are tracked: leisure time, work-related and transportation-related PAs. Questions about the frequency (weekly), duration (minutes), and intensity (moderate vs. vigorous) of physical exercise were included in the first two domains. In the last domain, there were questions to determine how many days a person typically exercises and how long they exercise each day [14]. For transportation-related PA, a metabolic equivalency score of 4.0 is advised, 4.0 for moderate-intensity activity, and 8.0 for vigorous-intensity activity, according to the NHANES guidelines. The following formula is used to translate PA into metabolic equivalent minutes per week (MET-min/week), which is based on weekly metabolic equivalent (MET) values for the type, frequency, and duration of the activity: PA (MET-min/wk) = MET × weekly frequency × duration of each PA [14]. Participants were categorized as meeting PA guidelines if they per week performed more than 150 min of moderate-intensity PA or 75 min of vigorous PA, or both (i.e. ≥600 MET-min/week) [15].

We formed four lifestyle groups according to DQ and PA based on prior research [13]: (1) persons with unhealthy diet and physically inactive, (2) those with healthy diet but physically inactive, (3) those with unhealthy diet but physically active, and (4) those with healthy diet and physically active. A healthy diet included those at or above the 60th percentile of the HEI [16]. In accordance with the PA Guidelines for Adults, respondents were considered active when they met the criterion of more than 600 MET-min/week moderate to vigorous PA [15].

### Assessment of mortality

Determined by correlating with the National Death Index as of February 25, 2019. The disease-specific death rates were obtained using the International Classification of Diseases-10 (ICD-10). We classified deaths into the following major cause of death categories: major cardiovascular diseases (ICD-10I00-I09, I11, I13, I20-I51) and all causes of death [17].

### Assessment of covariates

The study participants’ age, sex, body mass index (BMI), race, poverty-to-income ratio (PIR), education level, smoking status, alcohol consumption, medical history, and use of medications were among the pertinent data that the trained personnel collected. Race was categorized as Mexican American, non-Hispanic white, non-Hispanic black, or other races. PIR was divided into <1.0, or ≥1.0.Education is categorized as less than high school, high school, or more than high school. Smoking status was categorized into three groups: never smoker (referring to smoking less than 100 cigarettes in life), former smoker (referring to smoking more than 100 cigarettes in life and not smoking at all now), current smoker (referring to smoking more than 100 cigarettes in life and smoking some days or every day) [18]. Alcohol consumption was categorized as never, low to moderate (defined as drinking <3 drinks/day for females and <4 drinks/day for males), heavy (defined as ≥3 drinks/day for females and ≥4 drinks/day for males, or binge drinking [≥ 4 drinks on the same occasion for females, ≥ 5 drinks on the same occasion for males] on 5 or more days per month) [19]. Clinical indicators such as serum creatinine, serum uric acid, and the UACR were tested in the NHANES laboratory. A self-reported history of hypertension, a diastolic blood pressure of 90 mmHg or an systolic blood pressure of 140 mmHg, or the use of any antihypertensive medication were all regarded as indicators of hypertension [20]. A fasting glucose level above 7.0 mmol/L, an HbA1c level above 6.5%, the use of any antidiabetic medication, or a self-reported history of diabetes are the criteria used by the American Diabetes Association to diagnose diabetes [21]. Hyperlipidemia is defined as 200 mg/dL of total cholesterol, 150 mg/dL of triglycerides, 40 mg/dL of high-density lipoprotein for men and 50 mg/dL for women, or 130 mg/dL of low-density lipoprotein [22]. Alternatively, hyperlipidemia was also indicated by the use of cholesterol-lowering medications.

### Statistical analyses

Means and standard deviations were used to characterize regularly distributed data, whereas medians and interquartile ranges were used to characterize nonnormally distributed variables. To ascertain the statistical significance for each of the four groups, chi-square test analyses were conducted. Proportional outcome variables were expressed as proportions (%). Using Cox proportional risk regression models, corrected hazard ratios (HRs) and 95% confidence intervals (95% CIs) were computed and displayed. Model 1 was adjusted for age, sex, and race or ethnicity. Model 2 was further adjusted for the variables used in Model 1 as well as education, PIR, alcohol consumption, BMI, smoking status, total energy intake, serum creatinine, serum uric acid, UACR, eGFR, diabetes, hypertension, and hyperlipidemia. We generated Kaplan–Meier survival curves for all-cause and CVD mortality by lifestyle grouping. To consider any potential variability in the correlations of age, sex, and other demographic factors, exploratory subgroup analysis of the derived interactions was conducted. Furthermore, multiple sensitivity analyses were conducted to assess the resilience of our results.

The statistical significance level was set at *p* < 0.05. Every analysis was conducted using R version 3.4.3 (http://www.r-project.org, The R Foundation) and Empower software (www.empowerstats.com; X&Y Solutions, Inc., Boston, MA, USA).

## Results

### Subject characteristics

A total of 88.33% (*n* = 5,745) of the 6,504 people with CKD (mean age, 57.03 years) had at least a high school education, 50.26% (*n* = 3269) were female, 50.52% (*n* = 3286) were non-Hispanic white, and 19.68% (*n* = 1179) had household earnings below the federal poverty threshold. In total, 27.20% (1,769/6,504) of the participants scored at or above the 60th percentile on the HEI-2015, and 57.49% (3,739/6,504) of the responders met the PA guidelines. Additionally, a mean HEI-2015 score of 68.96 (SD 7.34) and a median duration of moderate to vigorous PA of 1,920 min per week were observed in people who followed a healthy diet and exercise regimen. A mean HEI-2015 score of 45.53 (SD 9.02) and a median duration of moderate-to-vigorous PA of 240 min per week were observed in individuals with poor diets and inactivity. Table 1 displays the baseline characteristics of the study population. Additionally, older males who were less likely to be obese, had never smoked, drank alcohol in moderation, and had high levels of education, household income and lower levels of kidney function were more likely to be among the participants who followed a good diet and exercise regimen.

**Table 1.** Baseline characteristics of the study participants.

		Lifestyle group				
Variable	Total	Unhealthy diet and physically inactive	Healthy diet but physically inactive	Unhealthy diet but physically active	Healthy diet and physically active	*P* value
Participants	6504	2020	745	2715	1024	
Age (years)	57.0 ± 20.3	54.7 ± 21.3	63.5 ± 18.8	54.6 ± 20.2	63.4 ± 16.9	<0.001
Sex, *n* (%)						<0.001
Female	3269 (50.3%)	1105 (54.7%)	402 (54.0%)	1267 (46.7%)	495 (48.3%)	
Male	3235 (49.7%)	915 (45.3%)	343 (46.0%)	1448 (53.3%)	529 (51.7%)	
Race or ethnicity, *n* (%)						<0.001
non-Hispanic White	3286 (50.5%)	1007 (49.9%)	421 (56.5%)	1312 (48.3%)	546 (53.3%)	
Mexican American	995 (15.3%)	343 (17.0%)	122 (16.4%)	400 (14.7%)	130 (12.7%)	
non-Hispanic Black	1377 (21.2%)	465 (23.0%)	132 (17.7%)	607 (22.4%)	173 (16.9%)	
Other races(including multiracial and other Hispanic)	846 (13.0%)	205 (10.2%)	70 (9.4%)	396 (14.6%)	175 (17.1%)	
Education, n (%)						<0.001
Less than high school	754 (11.6%)	252 (12.5%)	91 (12.2%)	291 (10.7%)	120 (11.7%)	
High school	2453 (37.7%)	799 (39.6%)	233 (31.3%)	1089 (40.2%)	332 (32.5%)	
More than high school	3292 (50.7%)	969 (48.0%)	421 (56.5%)	1332 (49.1%)	570 (55.8%)	
Poverty income ratio, n (%)						<0.001
Below poverty (<1.0)	1179 (19.7%)	383 (20.8%)	95 (13.6%)	568 (22.6%)	133 (14.3%)	
Above poverty (≥1.0)	4811 (80.3%)	1460 (79.2%)	603 (86.4%)	1949 (77.4%)	799 (85.7%)	
BMI (kg/m^2^), n (%)						0.023
<25	1903 (29.7%)	588 (29.6%)	244 (33.5%)	772 (28.7%)	299 (29.6%)	
≥25, <30	2052 (32.0%)	634 (32.0%)	222 (30.5%)	840 (31.3%)	356 (35.3%)	
≥30	2454 (38.3%)	762 (38.4%)	262 (34.0%)	1076 (40.0%)	354 (35.1%)	
Smoking status, n (%)						<0.001
Current smoker	1073 (17.6%)	395 (21.6%)	49 (6.8%)	534 (20.9%)	95 (9.5%)	
Former smoker	1996 (32.6%)	548 (30.0%)	288 (39.8%)	814 (31.8%)	346 (34.4%)	
Never smoker	3051 (49.9%)	887 (48.5%)	387 (53.5%)	1213 (47.4%)	564 (56.1%)	
Alcohol consumption, n (%)						<0.001
Never	817 (14.4%)	220 (13.0%)	102 (14.9%)	342 (14.5%)	153 (16.4%)	
Low to moderate	4003 (70.4%)	1186 (69.9%)	522 (76.0%)	1602 (67.8%)	693 (74.0%)	
Heavy	864 (15.2%)	291 (17.2%)	63 (9.2%)	420 (17.8%)	90 (9.6%)	
Total energy intake (kcal)	1949.6 ± 927.5	1948.2 ± 981.6	1751.1 ± 662.5	2038.7 ± 995.1	1860.8 ± 75	<0.001
Moderate to vigorous PA (MET-min/week)	819.0(252.0,2400.0)	240.0 (94.5–397.6)	226.0 (69.5–400.0)	1920.0 (1080.0–4800.0)	1920.0 (1036.0–3840.0)	<0.001
Total dietary quality score (HEI-2015)	51.8 ± 13.6	45.5 ± 9.0	69.2 ± 6.6	45.3 ± 9.3	69.0 ± 7.3	<0.001
eGFR, ml/min/1.73m^2^	74.3 (53.2–104.1)	77.6 (53.3–106.4)	60.5(51.5–92.5)	80.8(54.3–108.0)	61.4(51.9–94.1)	<0.001
UACR, mg/g	44.5 (21.3–96.4)	45.7 (30.8–100.8)	40.1 (11.8–84.5)	46.5 (30.3–101.7)	39.8 (11.0–85.0)	<0.001
Serum creatinine, umol/L (male)	101.7(79.6–123.8)	99.0 (79.6–123.8)	106.1 (86.9–114.9)	99.0 (79.6–120.2)	106.1(81.3–123.8)	0.018
Serum creatinine, umol/L (female)	72.5(61.9–96.4)	70.7 (61.9–93.7)	79.6 (61.9–97.2)	70.7 (58.3–92.8)	77.8(60.1–97.2)	0.002
Serum uric acid, umol/L	348.7 ± 97.2	347.6 ± 99.5	344.8 ± 92.8	351.1 ± 98.5	347.3 ± 92.1	0.433
Diabetes, n (%)	1940 (30.2%)	543 (27.3%)	233 (31.6%)	844 (31.4%)	320 (31.5%)	0.009
Hypertension, n (%)	3990 (61.4%)	1192 (59.0%)	511 (68.7%)	1623 (59.8%)	664 (64.8%)	<0.001
Hyperlipidemia, n (%)	4948 (76.1%)	1513 (74.9%)	576 (77.3%)	2038 (75.1%)	821 (80.2%)	0.004
Antidiabetic therapy, n (%)	1281 (19.7%)	345 (17.1%)	178 (23.9%)	548 (20.2%)	210 (20.5%)	<0.001
Antihypertensive therapy, n (%)	3247 (49.9%)	956 (47.3%)	432 (58.0%)	1289 (47.5%)	570 (55.7%)	<0.001
Antihyperlipidaemic therapy, n (%)	1863 (28.6%)	476 (23.6%)	234 (31.4%)	771 (28.4%)	382 (37.3%)	<0.001

Abbreviations: BMI: body mass index; PA: physical activity; MET: metabolic equivalent; HEI: Healthy Eating Index; eGFR: estimated glomerular filtration rate; UACR: urinary albumin to creatinine ratio.

### Associations between lifestyle groups and mortality

**Figure 2.**
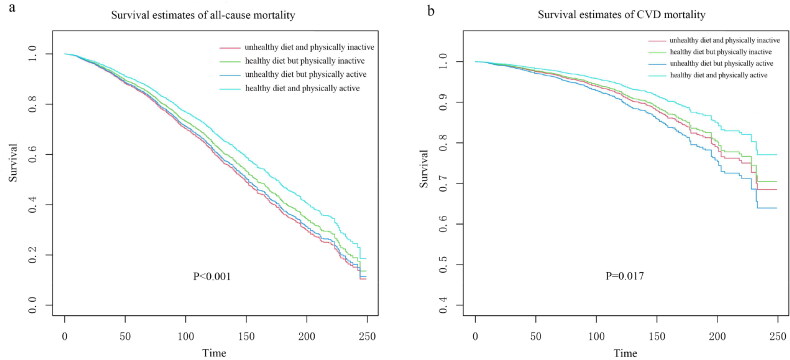
Kaplan-Meier survival curves for all-cause (a) and CVD (b) mortality of in CKD patients in four lifestyle groups (unhealthy diet and physically inactive, healthy diet but physically inactive, unhealthy diet but physically active, healthy diet and physically active).

During a median follow-up period of 111 months, 1,971 participants with CKD died from all-cause mortality, and 567 died from CVD. For the single effect of PA or DQ on mortality (Table 2), we noted that the single DQ effect was significantly and positively associated with mortality: CKD participants with higher DQ had a lower risk of all-cause mortality [0.83, 95% CI (0.74, 0.92)] and CVD mortality [0.73, 95% CI (0.60, 0.90)] than those with poor DQ. There was also a significant association between PA, when included as a continuous variable in the model, and all-cause mortality in CKD patients (*p* < 0.05). In addition, we explored the relationships between HEI-2015 components and all-cause mortality and CVD mortality. The results showed that according to the HEI-2015 component intake scale, higher scores of total fruits, whole fruits, and saturated fats were associated with lower all-cause mortality, and CVD mortality in patients with CKD, whereas higher scores of total protein foods, seafood and plant proteins were associated with lower CVD mortality but higher all-cause mortality (Supplementary Table 1). In relation to the combined effect of PA and DQ on CKD mortality, when comparing CKD mortality between lifestyle groups, the risk of all-cause mortality [0.75, 95% CI (0.64, 0.87)] and the risk of CVD mortality [0.69, 95% CI (0.51, 0.93)] were significantly lower in patients with CKD who had healthy diets in combination with recommended PA than in those who had unhealthy diets and low PA levels. Finally, we used Kaplan–Meier survival analysis to examine all-cause mortality and CVD mortality for various lifestyle combinations. The curves demonstrated that patients with healthy diets and recommended PA had the lowest lifetime risk of all-cause and CVD mortality (*p* < 0.001 and *p* = 0.017, respectively) (Figure 2).

**Table 2.** HRs For the associations of PA, DQ and their combined effects with all-cause and CVD mortality in the CKD population.

Exposure	Non-adjusted	Adjust I	Adjust II
**All-cause mortality**			
HEI-2015 score Continuous	1.01 (1.00, 1.01)<0.001	0.99 (0.98, 0.99)<0.001	0.99 (0.99, 0.99)<0.001
HEI-2015 score Categorical			
Lower DQ	1.0	1.0	1.0
Higher DQ	1.18 (1.07, 1.30)<0.001	0.74 (0.67, 0.81)<0.001	0.83 (0.74, 0.92) <0.001
Moderate to vigorous PA Continuous, MET-min/week	0.99 (0.99, 0.99)<0.001	0.99 (0.99, 0.99)0.003	0.99 (0.99, 0.99) 0.013
Moderate to vigorous PA Categorical			
Not met PA	1.0	1.0	1.0
Met PA	0.92 (0.84, 1.01)0.072	0.87 (0.79, 0.95)0.002	0.92 (0.83, 1.02) 0.109
Lifestyle group			
Unhealthy diet and physically inactive	1.0	1.0	1.0
Healthy diet but physically inactive	1.29 (1.13, 1.47)<0.001	0.84 (0.73, 0.96)0.010	0.88 (0.76, 1.03)0.106
Unhealthy diet but physically active	0.97 (0.87, 1.08)0.533	0.95 (0.85, 1.06)0.338	0.96 (0.85, 1.08) 0.521
Healthy diet and physically active	1.05 (0.91, 1.21)0.496	0.62 (0.54, 0.71)<0.001	0.75 (0.64, 0.87)<0.001
P for trend	0.801	<0.001	0.004
**CVD mortality**			
HEI-2015 score Continuous	1.00 (1.00, 1.01)0.378	0.98 (0.97, 0.99)<0.001	0.98 (0.98, 0.99)<0.001
HEI-2015 score Categorical			
Lower DQ	1.0	1.0	1.0
Higher DQ	1.06 (0.88, 1.27)0.556	0.64 (0.53, 0.78)<0.001	0.73 (0.60, 0.90)0.003
Moderate to vigorous PA Continuous, MET-min/week	0.99 (0.99, 0.99)0.042	1.00 (1.00, 1.00)0.671	1.00 (1.00, 1.00)0.982
Moderate to vigorous PA Categorical			
Not met PA	1.0	1.0	1.0
Met PA	1.04 (0.88, 1.22)0.677	0.98 (0.83, 1.15)0.764	1.03 (0.86, 1.24)0.722
Lifestyle group			
Unhealthy diet and physically inactive	1.0	1.0	1.0
Healthy diet but physically inactive	1.28 (0.99, 1.66)0.056	0.80 (0.62, 1.04)0.095	0.93 (0.70, 1.23)0.590
Unhealthy diet but physically active	1.15 (0.95, 1.40)0.153	1.12 (0.92, 1.37)0.248	1.18 (0.95, 1.47)0.135
Healthy diet and physically active	1.00 (0.77, 1.32)0.976	0.58 (0.44, 0.76)<0.001	0.69 (0.51, 0.93) 0.017
P for trend	0.538	0.024	0.353

Abbreviations: HRs: hazard ratios; CKD: chronic kidney disease; CVD: cardiovascular disease; PA: physical activity; DQ: dietary quality; HEI: Healthy Eating Index.

The unadjusted model was not adjusted.

Model I was adjusted for age, sex, and race or ethnicity.

Model II was adjusted for age, sex, race or ethnicity, education, PIR, alcohol consumption, BMI, smoking status, total energy intake, serum creatinine, serum uric acid, UACR, eGFR, diabetes, hypertension, and hyperlipidemia.

### Subgroup analyses and sensitivity analyses

In the stratified analysis of CKD populations with different lifestyles and all-cause mortality rates (Table 3), there were notable interactions between age and race subgroups, with older age groups and non-Hispanic Blacks showing stronger risk-positive effects on reducing both all-cause mortality and CVD mortality (*p* < 0.05 for interaction). Whereas no interaction was observed between different lifestyles and CVD mortality in different subgroups, the results were robust (Supplementary Table 2). The sensitivity analysis (Supplementary Table 3) revealed that there was no significant effect on reducing CVD mortality, but the benefit of a healthy lifestyle on reducing all-cause mortality persisted when we excluded participants with CVD. After further adjustment for underlying disease or medication history, the results did not change substantially.

**Table 3.** Associations Between different lifestyle groups and all-cause mortality among CKD patients in the stratified analysis.

	Lifestyle group							
Variable	Unhealthy diet and physically inactive	Healthy diet but physically inactive		Unhealthy diet but physically active		Healthy diet and physically active		P interaction
		Adjusted HR (95%CI)	*P* Value	Adjusted HR (95%CI)	*P* Value	Adjusted HR (95%CI)	*P* Value	
**Age**								0.007
<60	1[Reference]	0.65 (0.36, 1.17)	0.150	0.79 (0.58, 1.07)	0.126	1.28 (0.82, 2.01)	0.278	
≥60	1[Reference]	1.07 (0.91, 1.25)	0.404	0.99 (0.86, 1.12)	0.823	0.79 (0.67, 0.93)	0.006	
**Sex**								0.672
Female	1[Reference]	0.99 (0.78, 1.25)	0.919	0.86 (0.71, 1.05)	0.131	0.77 (0.60, 0.99)	0.038	
Male	1[Reference]	1.08 (0.88, 1.32)	0.449	0.95 (0.82, 1.11)	0.554	0.86 (0.70, 1.05)	0.138	
**Race or ethnicity**								0.032
non-Hispanic White	1[Reference]	1.13 (0.94, 1.35)	0.195	0.96 (0.83, 1.11)	0.568	0.88 (0.73, 1.06)	0.175	
Mexican American	1[Reference]	0.75 (0.45, 1.24)	0.260	0.78 (0.53, 1.17)	0.233	1.19 (0.76, 1.85)	0.441	
non-Hispanic Black	1[Reference]	0.85 (0.55, 1.30)	0.451	0.93 (0.69, 1.26)	0.654	0.44 (0.26, 0.76)	0.003	
Other races(including multiracial other Hispanic)	1[Reference]	1.10 (0.55, 2.17)	0.793	1.09 (0.63, 1.90)	0.751	0.60 (0.30, 1.20)	0.149	
**Education**								0.444
Less than high school	1[Reference]	0.88 (0.59, 1.33)	0.547	0.92 (0.68, 1.24)	0.581	0.71 (0.47, 1.06)	0.093	
High school	1[Reference]	1.02 (0.79, 1.32)	0.853	1.06 (0.89, 1.28)	0.509	1.01 (0.78, 1.31)	0.948	
More than high school	1[Reference]	1.12 (0.90, 1.40)	0.306	0.85 (0.70, 1.02)	0.086	0.77 (0.62, 0.97)	0.026	
**Poverty income ratio**								0.181
Below poverty (<1.0)	1[Reference]	0.94 (0.59, 1.51)	0.814	0.87 (0.65, 1.17)	0.365	0.50 (0.30, 0.84)	0.008	
Above poverty (≥1.0)	1[Reference]	1.05 (0.89, 1.23)	0.575	0.95 (0.83, 1.08)	0.420	0.88 (0.75, 1.04)	0.127	
**Smoking status**								0.332
Current smoker	1[Reference]	1.65 (1.02, 2.66)	0.043	1.00 (0.75, 1.33)	0.983	0.99 (0.59, 1.66)	0.971	
Former smoker	1[Reference]	1.04 (0.83, 1.30)	0.716	0.94 (0.78, 1.14)	0.532	0.89 (0.70, 1.13)	0.351	
Nonsmoker	1[Reference]	0.90 (0.72, 1.14)	0.388	0.94 (0.78, 1.14)	0.522	0.74 (0.59, 0.93)	0.011	
**Alcohol use**								0.680
Never	1[Reference]	0.83 (0.55, 1.25)	0.372	0.83 (0.60, 1.13)	0.236	0.76 (0.51, 1.13)	0.172	
Low to moderate	1[Reference]	1.07 (0.90, 1.27)	0.443	0.97 (0.85, 1.11)	0.681	0.87 (0.73, 1.04)	0.115	
Heavy	1[Reference]	1.48 (0.76, 2.87)	0.252	0.77 (0.51, 1.18)	0.229	0.69 (0.38, 1.26)	0.227	

Note: These hazard ratios are derived from the Cox regression of Model II.

## Discussion

Over one in seven individuals in the U.S. are thought to suffer from CKD, with an approximate number of 37 million individuals [23]. Kidney insufficiency, end-stage kidney disease (ESKD), and CVD can all develop from CKD over time. Complications associated with CKD may accelerate disease progression and increase the risk of cardiovascular-related disease. In the majority of healthy populations, a healthy lifestyle is recognized to be linked to a low risk of cardiometabolic disease and mortality [3]. However, there is a paucity of evidence on healthy lifestyles and mortality risk in patients with CKD. Based on our research of NHANES 1999–2018 data, we discovered that among US individuals with CKD, the lower the all-cause and CVD mortality rates were, the more PA and healthier dietary patterns the participants followed. When sensitivity analyses accounted for additional possible factors, the correlations remained significant. However, stratified analyses revealed that older age groups (≥60) and non-Hispanic Blacks were more positively impacted by the benefits of sustaining a healthy lifestyle in terms of lowering all-cause mortality.

In people with and without CKD, physical inactivity has been linked to higher death rates, according to research conducted in recent years. Peak oxygen consumption, self-reported physical function, and physical performance were significantly lower in CKD patients who did not require kidney replacement therapy as the eGFR decreased [24]. These findings were linked to frailty and disability, which may be caused by renal anemia, malnutrition, uremic toxins, vitamin D deficiency, and abnormalities in potassium metabolism [25]. However, current findings in studies of the positive effects of PA on outcomes in CKD patients are limited by their consistency [26]. Peak oxygen consumption, exercise duration, physical function, and overall health were significantly improved following aerobic training, according to a meta-analysis of 31 randomized controlled trials [27]. In contrast, Chen et al.’s cohort study revealed that increased PA did not significantly lower long-term mortality in patients with CKD [28]. This finding is consistent with the effect on mortality observed in our study when PA was used as a categorical variable, which may be a result of different study designs. Future research could examine the relationships of PA intensity, duration, and frequency with mortality, as our study examined only patterns related to PA. In addition, we jointly examined the effect of the Healthy Eating Index as a whole on CKD survival, which has not been previously reported.

Food is vital to life, and it contributes significantly to good health in addition to enabling survival. An increasingly significant factor influencing the ultimate clinical result of CKD is malnutrition. A high-quality plant-based diet may improve human biology and act as a medicine through a variety of processes, according to the KDOQI 2020 guidelines [29]. Furthermore, a high-quality plant-based diet may help with CKD comorbidities, and the consequences include infections, acute renal injury, and cardiovascular disease [30]. A high-quality diet rich in fruits, vegetables, whole grains, nuts, and legumes may be able to decrease inflammatory processes [31]. It has been suggested that high dietary acid load may increase kidney injury and CKD progression by elevating ammonium concentrations, causing complement activation, or by stimulating endothelin-1 and aldosterone production, leading to fibrosis [32], whereas fruits and vegetables with alkalizing potential can reduce the net dietary acid load [33]. Previous studies have proposed an association between healthy dietary patterns and higher survival in patients with CKD. In a meta-analysis of seven cohort studies, it was found that a healthy dietary pattern (rich in vegetables, fruits, legumes, whole grains, and fiber, and low in red meat, sodium, and refined sugars) was associated with a lower risk of death [6]. Our findings are largely consistent with those of previous studies, but in particular we found that higher scores of total protein foods were associated with lower CVD mortality but higher all-cause mortality. KDOQI suggests that the recommended protein intake should not only refer to the stage of CKD, but also take into account the metabolic status of the patient, whether there is a combination of diabetes mellitus and glycemic control [34]. This is mainly due to the potential risk of very low protein intake leading to malnutrition in CKD patients, who have a higher mortality rate [35]. Although there is still disagreement on the recommendations for protein supply, a more unified understanding is that CKD patients should adopt an appropriate protein diet. Individualized nutritional programs should be developed under the guidance of a professional dietitian or equivalent, and their implementation should be monitored, evaluated, and adjusted in a timely manner. The association between patients’ total protein intake score and risk of death in this study only provides a direction that needs to be explored by further studies in the future.

To further illustrate the cobenefits of sufficient PA and a nutritious diet on lowering the risk of death in US individuals with CKD, we assessed health markers in each of the four lifestyle groups. Table 2 shows that the associations of the two lifestyles—a healthy diet combined with physical inactivity and an unhealthy diet combined with recommended PA—with the risk of death were not statistically significant. This finding shows that both PA and DQ together may be more useful than either one alone in lowering the risk of death in CKD patients. Therefore, it may be assumed that the combination of maintaining a good diet and engaging in frequent PA prevents patients with CKD from experiencing early death, and neither should be disregarded. We also discovered that, in comparison to other races or ethnic groups, black Americans are generally reported to have lower DQ and the greatest incidence of diet-related disorders due to the influence of racial or ethnic disparities on lifestyle, which is consistent with our finding that maintaining a healthy lifestyle has a stronger positive effect on reducing all-cause and CVD mortality in non-Hispanic blacks [36]. According to these results, to achieve the best survival gains from CKD prevention methods, incorporating both DQ and PA is critical. These initiatives should be implemented both nationally and locally.

Nonetheless, this study still has several shortcomings. First, the primary constraint of this research is recall bias and misinterpretation problems due to the self-reported nature of the PA data. Second, it is possible that the participants underwent behavioral changes during the follow-up period, and the impact of these changes on risk estimates could not be accurately estimated. Furthermore, a few NHANES participants were disqualified because of incomplete data, which could have impacted the outcomes. Finally, participants’ vital status was assessed by matching the cohort to the National Death Index. Owing to the limited availability of NHANES survival data, we were unable to exclude patients who died within 2 years of follow-up, a limitation that may have biased our results.

## Conclusion

In this consecutive survey based on the 1999–2018 cycle for representative US adults with CKD, our research revealed that all-cause and CVD mortality in individuals with CKD was inversely associated with greater levels of PA and improved DQ. This underscores the importance of improving adherence to healthy eating patterns and recommended PA in preventing premature deaths among CKD patients, and offers policymakers a helpful resource to launch pertinent preventative initiatives and publicize them.

## Supplementary Material

Supplementary Table 3.docx

Supplementary Table 1.docx

Supplementary Table 2.docx

## Data Availability

The datasets analyzed during the current study are available on the NHANES official website, https://wwwn.cdc.gov/Nchs/Nhanes/.
