# Supplementary material for: Associations of combined physical activity and dietary quality with all-cause and cardiovascular disease mortality among US adults with chronic kidney disease
Source: Ren Fail. 2024 Dec 10;46(2):2437120. doi: 10.1080/0886022X.2024.2437120 (PMC11633433; doi:10.1080/0886022X.2024.2437120)
Supplement: Supplementary Table 3.docx [file IRNF_A_2437120_SM6238.docx]

**TableS3** Association of lifestyle with mortality with the exclusion of history of CVD participants, and adjustment for underlying medication history.

| **variable** | **Lifestyle group** | | | | | | |
| --- | --- | --- | --- | --- | --- | --- | --- |
|  | **Unhealthy diet and physically inactive** | **Healthy diet but physically inactive** | | **Unhealthy diet but physically active** | | **Healthy diet and physically active** | |
|  |  | **Adjusted HR (95%CI)** | ***P* Value** | **Adjusted HR (95%CI)** | ***P* Value** | **Adjusted HR (95%CI)** | ***P* Value** |
| **Excluding participants with presence of CVD** |  |  |  |  |  |  |  |
| All-cause mortality | 1[Reference] | 0.85 (0.71, 1.03) | 0.100 | 0.90 (0.77, 1.04) | 0.143 | 0.69 (0.57, 0.84) | <0.001 |
| CVD mortality | 1[Reference] | 0.87 (0.58, 1.29) | 0.485 | 1.21 (0.90, 1.63) | 0.198 | 0.71 (0.47, 1.07) | 0.099 |
| **Whether taking antiDiabetic、antiHypertensive or antiHyperlipidaemic medications** |  |  |  |  |  |  |  |
| All-cause mortality | 1[Reference] | 0.88 (0.76, 1.03) | 0.1069 | 0.96 (0.85, 1.08) | 0.510 | 0.75 (0.64, 0.87) | <0.001 |
| CVD mortality | 1[Reference] | 0.93 (0.70, 1.23) | 0.595 | 1.17 (0.94, 1.46) | 0.152 | 0.69 (0.51, 0.94) | 0.017 |

Abbreviations: CVD: cardiovascular disease.

The multivariable model was adjusted for age, sex, race or ethnicity, education, PIR, alcohol consumption, BMI, smoking status, total energy intake, serum creatinine, serum uric acid, UACR, eGFR, diabetes, hypertension, and hyperlipidaemia.
