# Supplementary material for: Associations of combined physical activity and dietary quality with all-cause and cardiovascular disease mortality among US adults with chronic kidney disease
Source: Ren Fail. 2024 Dec 10;46(2):2437120. doi: 10.1080/0886022X.2024.2437120 (PMC11633433; doi:10.1080/0886022X.2024.2437120)
Supplement: Supplementary Table 1.docx [file IRNF_A_2437120_SM6237.docx]

**Table S1** Association between HEI-2015 components and all-cause and CVD mortality in the CKD population.

| **Variables** | **All-cause mortality** |  |  | **CVD mortality** |  |
| --- | --- | --- | --- | --- | --- |
|  | **Adjusted HR（95%CI）** | **P Value** |  | **Adjusted HR（95%CI）** | **P Value** |
| Total vegetables | 0.98 (0.97, 1.00) | 0.106 |  | 0.97 (0.94, 1.01) | 0.117 |
| Greens and beans | 1.00 (0.99, 1.02) | 0.674 |  | 0.96 (0.93, 0.99) | 0.010 |
| Total fruits | 0.95 (0.93, 0.96) | <0.001 |  | 0.96 (0.93, 0.99) | 0.009 |
| Whole fruits | 0.94 (0.92, 0.95) | <0.001 |  | 0.96 (0.93, 0.99) | 0.003 |
| Whole grains | 1.01 (1.00, 1.02) | 0.026 |  | 0.99 (0.98, 1.01) | 0.494 |
| Dairy | 0.99 (0.98, 1.00) | 0.203 |  | 0.99 (0.97, 1.01) | 0.407 |
| Total protein foods | 1.04 (1.01, 1.06) | 0.003 |  | 0.95 (0.91, 0.99) | 0.047 |
| Seafood and plant proteins | 1.02 (1.00, 1.03) | 0.011 |  | 0.94 (0.92, 0.97) | <0.001 |
| Fatty acids ratio | 1.00 (0.99, 1.00) | 0.288 |  | 0.98 (0.97, 1.00) | 0.054 |
| Sodium | 0.99 (0.98, 1.00) | 0.120 |  | 1.01 (0.99, 1.03) | 0.322 |
| Refined grains | 1.01 (1.00, 1.02) | 0.042 |  | 1.00 (0.98, 1.02) | 0.813 |
| Saturated fats | 0.98 (0.97, 0.99) | <0.001 |  | 0.97 (0.95, 0.99) | 0.001 |
| Added sugars | 1.01 (1.00, 1.02) | 0.023 |  | 0.99 (0.97, 1.01) | 0.271 |

Abbreviations: HEI: Healthy Eating Index; CVD: cardiovascular disease; CKD: chronic kidney disease.

The multivariable model was adjusted for age, sex, race or ethnicity, education, PIR, alcohol consumption, BMI, smoking status, total energy intake, serum creatinine, serum uric acid, UACR, eGFR, diabetes, hypertension, hyperlipidaemia.
