# Supplementary material for: Associations of combined physical activity and dietary quality with all-cause and cardiovascular disease mortality among US adults with chronic kidney disease
Source: Ren Fail. 2024 Dec 10;46(2):2437120. doi: 10.1080/0886022X.2024.2437120 (PMC11633433; doi:10.1080/0886022X.2024.2437120)
Supplement: Supplementary Table 2.docx [file IRNF_A_2437120_SM6236.docx]

**Table S2** Associations between different lifestyle groups and CVD mortality among CKD patients in the stratified analysis.

| **variable** | **Lifestyle group** | | | | | | | **P interaction** |
| --- | --- | --- | --- | --- | --- | --- | --- | --- |
|  | **Unhealthy diet and physically inactive** | **Healthy diet but physically inactive** | | **Unhealthy diet but physically active** | | **Healthy diet and physically active** | |  |
|  |  | **Adjusted HR (95%CI)** | ***P* Value** | **Adjusted HR (95%CI)** | ***P* Value** | **Adjusted HR (95%CI)** | ***P* Value** |  |
| **Age** |  |  |  |  |  |  |  | 0.102 |
| <60 | 1[Reference] | 0.45 (0.09, 2.22) | 0.326 | 1.76 (0.95, 3.27) | 0.073 | 0.29 (0.04, 2.19) | 0.228 |  |
| ≥60 | 1[Reference] | 1.12 (0.84, 1.50) | 0.448 | 1.11 (0.88, 1.40) | 0.388 | 0.77 (0.56, 1.05) | 0.100 |  |
| **Sex** |  |  |  |  |  |  |  | 0.347 |
| Female | 1[Reference] | 0.85 (0.53, 1.34) | 0.479 | 0.87 (0.59, 1.28) | 0.470 | 0.59 (0.35, 0.98) | 0.043 |  |
| Male | 1[Reference] | 1.18 (0.82, 1.70) | 0.370 | 1.26 (0.96, 1.66) | 0.092 | 0.82 (0.56, 1.21) | 0.321 |  |
| **Race or ethnicity** |  |  |  |  |  |  |  | 0.084 |
| non-Hispanic White | 1[Reference] | 1.18 (0.84, 1.64) | 0.346 | 1.17 (0.90, 1.52) | 0.240 | 0.82 (0.58, 1.17) | 0.283 |  |
| Mexican American | 1[Reference] | 1.23 (0.41, 3.72) | 0.714 | 1.71 (0.78, 3.75) | 0.177 | 1.42 (0.53, 3.80) | 0.488 |  |
| non-Hispanic Black | 1[Reference] | 1.09 (0.51, 2.30) | 0.831 | 1.08 (0.62, 1.88) | 0.776 | 0.43 (0.16, 1.18) | 0.102 |  |
| Other races（including multiracial other Hispanic） | 1[Reference] | 0.37 (0.07, 1.90) | 0.232 | 0.19 (0.04, 0.88) | 0.034 | 0.09 (0.01, 0.62) | 0.015 |  |
| **Education** |  |  |  |  |  |  |  | 0.143 |
| Less than high school | 1[Reference] | 0.60 (0.24, 1.50) | 0.275 | 1.45 (0.86, 2.43) | 0.160 | 0.58 (0.24, 1.37) | 0.212 |  |
| High school | 1[Reference] | 1.41 (0.90, 2.20) | 0.133 | 1.16 (0.81, 1.65) | 0.417 | 1.14 (0.71, 1.83) | 0.599 |  |
| More than high school | 1[Reference] | 0.96 (0.63, 1.48) | 0.863 | 1.03 (0.73, 1.45) | 0.877 | 0.62 (0.39, 0.98) | 0.043 |  |
| **Poverty income ratio** |  |  |  |  |  |  |  | 0.649 |
| Below poverty (<1.0) | 1[Reference] | 0.59 (0.20, 1.78) | 0.352 | 0.93 (0.53, 1.62) | 0.793 | 0.56 (0.22, 1.43) | 0.229 |  |
| Above poverty (≥1.0) | 1[Reference] | 1.16 (0.86, 1.56) | 0.334 | 1.21 (0.95, 1.54) | 0.117 | 0.82 (0.59, 1.13) | 0.223 |  |
| **Smoking status** |  |  |  |  |  |  |  | 0.764 |
| Current smoker | 1[Reference] | 1.11 (0.36, 3.41) | 0.857 | 1.42 (0.83, 2.45) | 0.201 | 0.40 (0.09, 1.75) | 0.224 |  |
| Former smoker | 1[Reference] | 1.06 (0.69, 1.62) | 0.807 | 1.15 (0.81, 1.62) | 0.426 | 0.86 (0.54, 1.38) | 0.542 |  |
| Nonsmoker | 1[Reference] | 0.96 (0.63, 1.46) | 0.847 | 1.05 (0.74, 1.49) | 0.780 | 0.65 (0.42, 1.02) | 0.060 |  |
| **Alcohol use** |  |  |  |  |  |  |  | 0.246 |
| Never | 1[Reference] | 0.73 (0.32, 1.64) | 0.439 | 0.84 (0.47, 1.49) | 0.547 | 0.58 (0.27, 1.24) | 0.157 |  |
| Low to moderate | 1[Reference] | 1.12 (0.82, 1.54) | 0.464 | 1.16 (0.90, 1.49) | 0.243 | 0.83 (0.59, 1.16) | 0.277 |  |
| Heavy | 1[Reference] | 4.72 (1.00, 22.19) | 0.050 | 2.22 (0.84, 5.86) | 0.109 | 0.48 (0.09, 2.58) | 0.390 |  |

Notes: These hazard ratios are derived from the Cox regression of Model II.
